# A Case of Multifactorial Diabetic Ketoacidosis Acquired in the Intensive Care Unit: A Case Report

**DOI:** 10.7759/cureus.5128

**Published:** 2019-07-12

**Authors:** Ceressa T Ward, Babar Fiza, Amit Prabhakar, Gaurav Budhrani, Vanessa Moll

**Affiliations:** 1 Miscellaneous, Emory University Hospital Midtown, Atlanta, USA; 2 Anesthesiology, Emory School of Medicine, Atlanta, USA

**Keywords:** diabetic ketoacidosis, intensive care unit (icu), hyperglycemia

## Abstract

Diabetic ketoacidosis (DKA) is a potentially fatal endocrine emergency resulting from uncontrolled diabetes mellitus (DM). The development of DKA has been linked to a number of precipitating factors such as infectious process, ischemia, medications, and other medical-surgical illnesses. These factors have been found to aggravate or unmask pre-existing glucose dysregulation secondary to absolute or relative insulin deficiency and increased levels of counter-regulatory hormones. We describe the case of a 61-year-old male with a history of insulin dependent DM who develops DKA postoperatively after a three-vessel coronary artery bypass surgery and mitral valve repair while in the intensive care unit (ICU). The patient’s postoperative course was complicated by presumed pneumonia and hyperactive delirium. On postoperative day (POD) five, the patient’s insulin infusion was held due to non-symptomatic hypoglycemia. Eleven hours later, the insulin infusion was resumed to treat DKA after laboratory findings revealed hyperglycemia, an elevated β-hydroxybutyrate, and anion gap metabolic acidosis. Multiple contributing factors for the development of DKA are suspected and discussed. It is paramount that clinicians are knowledgeable of the multiple factors that can contribute to the development of DKA in the ICU.

## Introduction

Diabetic ketoacidosis (DKA) is a potentially fatal endocrine emergency which acutely develops from precipitating factors which can aggravate or unmask pre-existing glucose dysregulation. In addition to absolute or relative insulin deficiency, increased levels of counter-regulatory hormones (e.g., catecholamines, cortisol, glucagon, and growth hormone) promote hyperglycemia by increasing gluconeogenesis, glycogenolysis and decreasing insulin sensitivity in peripheral tissues. This leads to lipolysis and subsequent elevations in free fatty acids which then undergo hepatic oxidation to ketone bodies (β-hydroxybutyrate and acetoacetate). Accumulation of these ketone bodies results in ketonemia and anion gap metabolic acidosis [1-3]. 

In up to 40% of DKA cases, known diabetics experienced episodes of extreme physiologic stress secondary to an infectious process, ischemia, or other medical-surgical illnesses. The lack of exogenous insulin due to medical noncompliance, inadequate dosing, and in some cases, malfunction of insulin pumps accounts for an estimated 21% to 49% of DKA cases. Medications that alter carbohydrate metabolism, anorexia, and endocrine disorders have also been implicated as risk factors for DKA. Notably, up to 10% of DKA cases are described as idiopathic [1-3]. Although DKA patients are typically managed in general care areas, those with severe altered mental status or concomitant critical illness are admitted to the intensive care unit (ICU) [1].

Here we describe a case of DKA precipitated by multiple factors in the ICU in a known insulin-dependent diabetic patient on postoperative day (POD) five after a three-vessel coronary artery bypass graft (CABG) and mitral valve repair (MVr). Multiple cases of euglycemic DKA developing in the ICU secondary to singular factors such as pregnancy or use of sodium-glucose cotransporter 2 inhibitors have been previously reported in the literature [4]. However, to our knowledge, this case represents the first description of multifactorial DKA acquired in an ICU.

## Case presentation

A 61-year-old male with a past medical history of a cerebrovascular accident with residual left-sided deficit, hypertension, hyperlipidemia, and type 2 diabetes mellitus (DM) managed on an insulin pump with a perioperative hemoglobin A1c (HbA1c) of 7.2% (normal range: 4.3%-6.1%) underwent a three-vessel CABG and MVr with annuloplasty ring. The patient was weaned off vasopressors by POD four yet continued on a low dose of inotrope. Hydrocortisone was started, empirically, in the immediate postoperative period for circulatory shock presumably due to relative adrenal insufficiency. This therapy was discontinued on the morning of POD five.

Given the presence of fever and infiltrates on chest X-ray, cefepime and vancomycin were initiated as empiric coverage for a suspected left lower lobe pneumonia on POD four. Of note, blood cultures remained no growth to date. The patient was also started on oral quetiapine 25 mg twice daily for acute hyperactive delirium. The patient’s blood glucose (BG), prior to quetiapine initiation, ranged from 79-206 mg/dL (averaging the mid-100s; normal range 65-100 mg/dL). Our cardiothoracic surgery (CTS) insulin infusion protocol, with specific blood glucose (BG) goals of 110-140 mg/dL, is typically active until POD two before it is transitioned to sliding scale with or without a scheduled insulin regimen for extubated patients who are able to tolerate a diet. Diabetics that typically manage their BG with an insulin pump are permitted to resume use of this device given they have the cognition to manipulate the settings for insulin administration based on point of care (POC) BG readings. However, this patient’s altered mentation prohibited safe inpatient use of his insulin pump and thus necessitated the continuation of an insulin infusion. Due to the patient's documented dysphagia, enteral nutrition via a nasogastric tube was continued after he was extubated.

**Figure 1:**
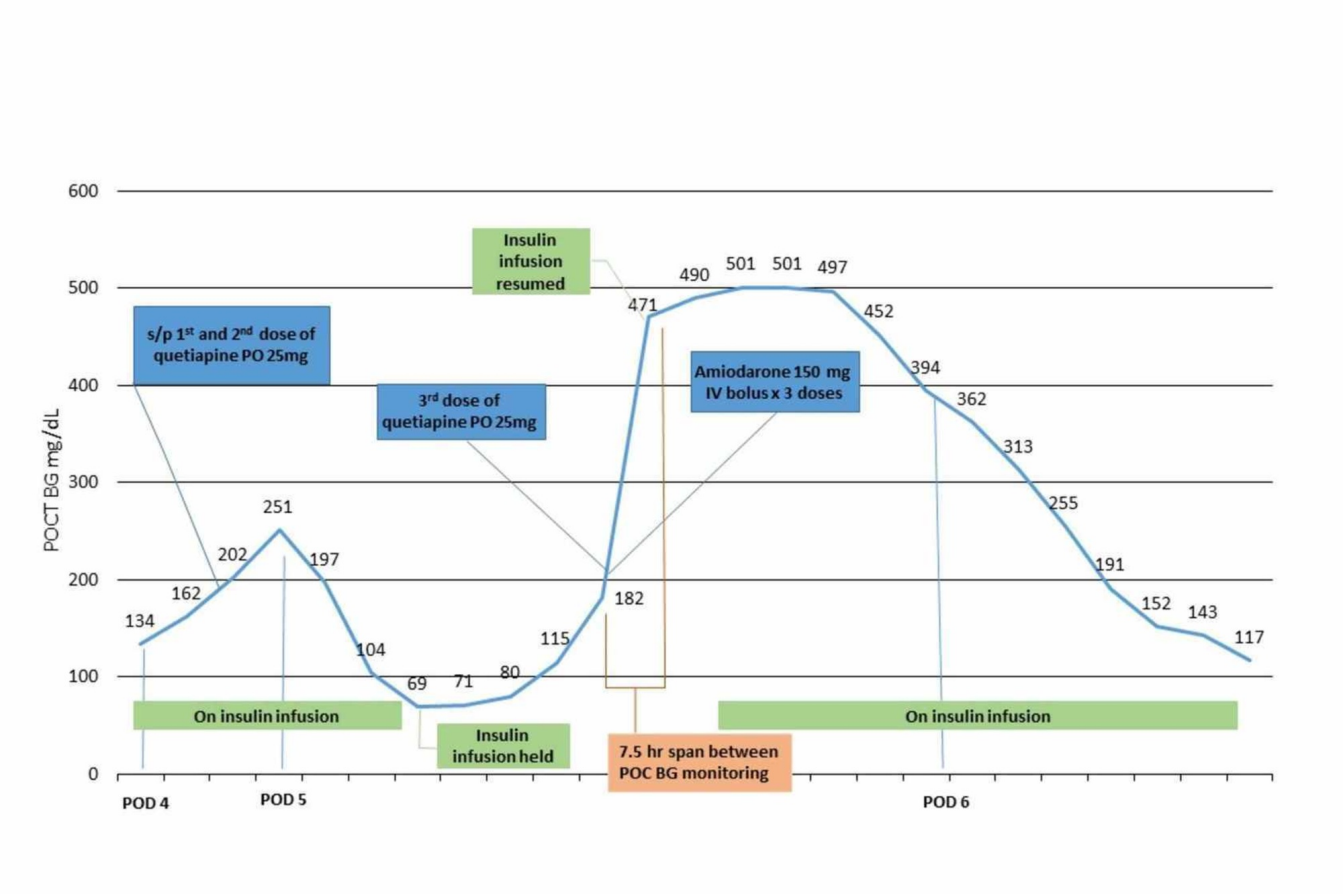
Clinical Timeline

On the morning of POD five, the insulin infusion was held secondary to non-symptomatic hypoglycemia with a POC BG of 69 mg/dL. Quetiapine therapy was discontinued after the third dose secondary to lethargy. Small doses of intravenous (IV) haloperidol on an as needed basis for agitation were utilized instead. The patient received one dose of IV haloperidol 1 mg during the early evening of POD five and was later started on a low dose dexmedetomidine infusion for persistent agitation. That same evening, the patient developed atrial fibrillation with rapid ventricular response. This was managed with three 150 mg boluses of IV amiodarone along with a short amiodarone infusion (Figure 1).

Approximately 7.5 hours after the last POC BG of 182 mg/dL, laboratory findings on POD five revealed both serum and POC BG levels ranging from 452 mg/dL to greater than 500 mg/dL, a β-hydroxybutyrate of 1.8 (normal range: ≤0.3), an anion gap of 17 mmol/L, and a lactate of 4 mmol/L thus suggesting a diagnosis of DKA. Enteral feedings were discontinued. DKA was managed with four liters of balanced crystalloids for fluid resuscitation, two boluses of regular insulin IV (totaling 35 units), and re-initiation of the insulin infusion that had been held for 11 hours given the previous hypoglycemic episode (Figure 1). At the time of the DKA diagnosis, the patient had a phosphorus level of 1.1 mg/dL (normal range: 2.4 -4.7 mg/dL) which was repleted with 20 mmol of IV sodium phosphate. Seven hours after the initial diagnosis, the anion gap was 7 mmol/L and DKA resolved (Table 1).

**Table 1:** Diabetic Ketoacidosis (DKA) Clinical Diagnostic Data WBC: white blood cell; BHB: β-hydroxybutyrate

	Carbon Dioxide (mmol/L)	Phosphorus (mg/dL)	Anion Gap (mmol/L)	pH	Lactate (mmol/L)	WBC^*^ (10^3^/mcL)	BHB^+^ (mmol/L)
POD 5 @ 2025	17 (L)	1.6 (L)	17 (H)			13.3 (H)	
POD 5 @ 2231	20 (L)	1.1 (L)				13.1 (H)	1.8 (H)
POD 5 @ 2305				7.41	4 (H)		
POD 6 @ 0412	29	1.5 (L)	7			11.9 (H)	
POD 6 @ 0932	25.1	>2		7.47	1.77		

An endocrine consult note on POD six suggested that DKA was likely secondary to the amiodarone boluses administered hours earlier. The patient remained clinically stable on an insulin infusion before being transitioned to a basal/bolus insulin regimen on POD 16 in preparation for discharge to cardiac rehab. The patient’s acute delirium continued to be managed with scheduled IV haloperidol 1 mg and a retrial of quetiapine PO without incident. The patient was eventually discharged home from inpatient cardiac rehab on a basal/bolus insulin regimen.

## Discussion

**Figure 2:**
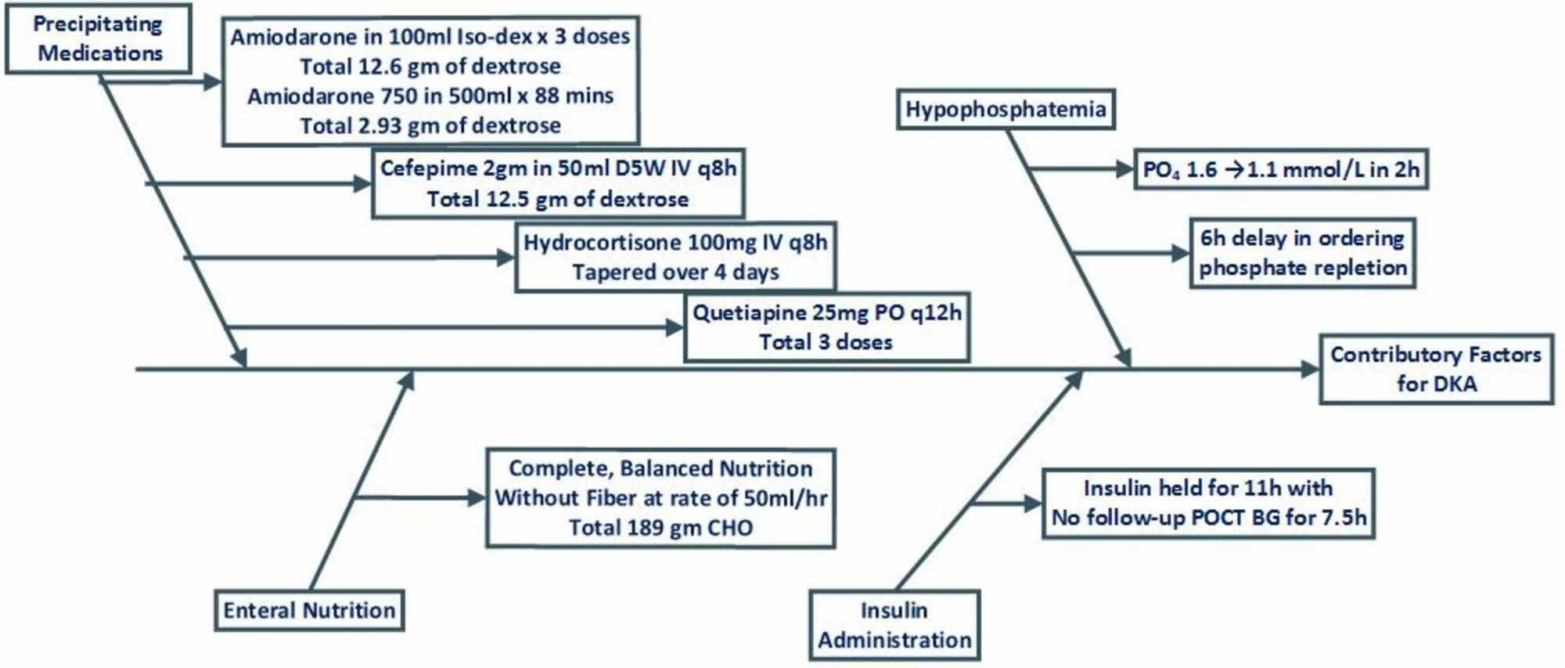
Contributing Factors for Diabetic Ketoacidosis (DKA)

DKA may be precipitated by multiple factors impacting glucose regulation. In this particular case, the authors believe that DKA manifested from a combination of physiologic stress, medications, nonadherence to insulin protocol, and electrolyte imbalance. While infection is a common cause of DKA, our patient was on day two of empiric antibiotics at the time of the DKA episode [1-2]. Thus, we consider other precipitating factors to be a strong possibility (Figure 2).

Stress hyperglycemia is an expected phenomenon which can occur up to 21 days after CTS [5] however, there is limited data depicting the development of DKA in the immediate postoperative period. In 2016, Haldar described the case of a 52-year-old female with no known history of DM who developed DKA approximately 30 minutes status-post a laminectomy and tumor excision under general anesthesia. The patient received no steroids or dextrose-containing fluids, intraoperatively. The authors concluded that surgical stress and anesthesia activated the patient’s dormant DM [6]. At our institution, all CTS patients are initiated on a continuous insulin infusion protocol until POD two. After this time, patients with known DM or those with higher insulin requirements are transitioned to a basal/bolus insulin regimen. In efforts to prevent postoperative deep sternal wound infections, insulin therapy is adjusted to maintain a target BG range of 110-140 mg/dL [5]. In this particular case, transitioning back to an insulin pump would have been ideal, however, this was delayed until supplies were available and the patient’s cognition would allow him to safely manage the device, independently.

In addition to physiological stress, numerous reports have implicated atypical antipsychotics (AA) as contributing factors for DKA manifestation [7]. Although the mechanism for AA-induced DKA is unknown, it is speculated that AAs antagonize M3 muscarinic receptors thus decreasing insulin secretion [7]. A number of AA-induced cases have been reported in patients with no previous history of DM. After DKA resolution, patients were discharged with either no treatment, diet control, or continued on hypoglycemic medications; in one case, medications were eventually discontinued. Another case series reported 17 DKA fatalities which were attributable to AA most notably clozapine and olanzapine [8]. However, Madsen reported the case of a young male on quetiapine who presented with complaints of polydipsia, vomiting, and upper abdominal pain. Twelve hours later, the patient deteriorated as he became obtunded, hypoxic, acidotic (pH 7.18), and was in hypovolemic shock with a lactate of 4.5 mmol/L. The patient was treated with volume resuscitation, insulin infusion, and vasoactive therapy. He later died of multi-organ failure [9]. Given a 7.25%-13% rate of mortality from AA-induced DKA [7,10] and the increased use of AA to manage ICU delirium, as seen in our patient, heightened awareness of this causal link is imperative.

Seen as early as four days, AA-induced DKA can occur at any time following the initiation of medication. Risk factors include male gender, age 30-60 years, history of DM, history of schizophrenia, type of AA (e.g., clozapine, olanzapine or quetiapine), polypharmacy, and ethnicity (African or Caucasian descent) while weight gain is an independent variable [7-8,10]. Our patient was a Caucasian male with a history of DM, receiving quetiapine, and 61 years old at the time of presentation.

Although amiodarone-induced DKA has not been reported, hyperglycemia secondary to amiodarone-induced dysregulation of carbohydrate metabolism has been previously described in multiple case reports involving both children and adults. Patients were noted to have normal baseline thyroid studies and no documentation of thyroid dysfunction. Alterations in glucose metabolism were reported to occur as early as five hours after initiation of amiodarone while in some cases manifestations of dysglycemia were delayed up to one year. In all cases, BG normalized after amiodarone was discontinued; only one patient was unable to withdraw from antihyperglycemic therapy [11-13]. One report suggests that amiodarone-induced hyperglycemia may be the effect of dysregulation in carbohydrate metabolism due to alterations in neurohormonal stimulation or organ toxicity (e.g., hepatic or pancreatic) [11]. A small prospective, non-randomized trial of 10 non-diabetic patients receiving amiodarone attempted to invalidate the theory that amiodarone impacted glucose tolerance. While a statistically significant rise in HbA1c was noted at six months when compared to baseline, there was no statistically significant difference in HbA1c at nine months compared to baseline [14]. The small sample of this study limits the ability to unequivocally rule out amiodarone as a potential contributing factor for hyperglycemia in our patient.

The absence or insufficient provision of insulin therapy is the second most common precipitating cause for DKA [1-3]. After a non-symptomatic episode of hypoglycemia (i.e., POC BG 69 mg/dL), our patient’s insulin infusion was held. Approximately three hours later, a repeat POC BG of 182 mg/dL was documented and per protocol, the insulin infusion should have been resumed at 50% of the prior infusion rate. For reasons, unbeknownst to the authors, the insulin infusion was not resumed and the hourly POC BG were not documented as required. Nearly seven and a half hours later, a repeat POC BG of 471 mg/dL necessitated the re-initiation of the insulin infusion after being held for 11 hours. The improvements in technology of insulin pumps after 1993, along with patient education, was cited as a contributing factor to the reduction of DKA in patients utilizing the pumps [2]. Due to his ongoing hyperactive delirium, the patient’s insulin pump had not been reconnected. 

Osmotic diuresis secondary to DKA can result in severe electrolyte depletion, particularly hypophosphatemia. In addition, a case series identified a plethora of reasons leading to hypophosphatemia in CTS patients. The inverse relationship between hypophosphatemia and hyperglycemia in non-diabetic patients has been previously documented [15]. It has been postulated that the development of insulin insensitivity may be due to ATP inactivation which is needed for energy metabolism [16]. Notably, the authors revealed that phosphate nadirs of <0.5-2 mmol/L yielded maximal insulin infusion rates of 15-73 units/hour in this population. When phosphate levels more than doubled, insulin requirements declined significantly to 0-8 units/hour [16]. Similarly, our patient’s insulin requirements declined from 12.6 to 6 units/hour with the infusion of sodium phosphate. By the time hypophosphatemia had normalized to 2.8 mmol/L, insulin requirements declined further to 1 unit/hour. At the time of DKA diagnosis, our patient was hypophosphatemic with a phosphorus level of 1.6 mg/dL and required 5 units/hr of insulin which was rapidly titrated to 12.6 units/hr with a corresponding phosphorus level of 1.1 mg/dL. After the patient received a six-hour infusion of sodium phosphate 20 mmol, the insulin infusion was weaned to 2 units/hr and the phosphorus level was 2.8 mg/dL.

## Conclusions

The development of DKA in an ICU is an unusual phenomenon. Generally, patients are admitted to the ICU for DKA management given the potential complications related to severe cases. This case presents multiple contributory factors for the development of DKA, in the ICU, in a diabetic patient status-post CABG and MVr. Furthermore, this case highlights the fact that DKA can develop in the critical care setting and critical care practitioners need to be vigilant at recognizing these, and other, potential contributory factors for DKA.
